# Thyroid psychosis: when your hormones take over your mind: a case report

**DOI:** 10.1192/j.eurpsy.2024.361

**Published:** 2024-08-27

**Authors:** K. Razki, C. Najar, U. Ouali, S. Ben Aissa, R. Jomli

**Affiliations:** ^1^Razi Hospital, LaManouba, Tunisia; ^2^Psychiatry A, Razi Hospital, LaManouba, Tunisia

## Abstract

**Introduction:**

Hyperthyroidism, characterized by excessive production of thyroid hormones, is a common endocrine disorder that affects various body systems. While most commonly recognized for its classic symptoms such as weight loss, tremors, and palpitations, it is important to acknowledge that hyperthyroidism can also lead to a rare but significant complication: psychosis. Psychosis in the context of hyperthyroidism refers to the presence of delusions, hallucinations, and disordered thinking, which can significantly impact an individual’s mental health and overall well-being.

**Objectives:**

This case report aims to describe a rare case of hyperthyroidism-related psychosis in a patient including the clinical presentation, diagnosis, and management. Additionally, we aim to increase awareness of and promote further research into this condition.

**Methods:**

We present a comprehensive case report detailing the clinical course of a 29-year-old male patient with no previous medical or psychiatric history, who sought urgent psychiatric evaluation at the Razi Hospital La Manouba’s emergency department due to escalating symptoms of agitation and paranoia persisting for three days. The patient, identified as Mr. S.O., a Tunisian male, presented with severe agitation and paranoia necessitating the use of restraints upon admission to the psychiatric emergency department. The initial physical examination revealed no notable abnormalities, except for the presence of tachycardia, which was subsequently confirmed on an electrocardiogram, arousing suspicion of a primary psychiatric illness.

**Results:**

While the standard blood workup yielded unremarkable findings, the endocrine workup revealed decreased levels of thyroid-stimulating hormone (TSH) and elevated free thyroxine (FT4). Further laboratory investigations demonstrated elevated anti-thyroid-stimulating hormone receptor antibodies, leading to the diagnosis of Graves’ disease. Collaborative consultation with an endocrinologist resulted in the initiation of a treatment regimen consisting of methimazole, propranolol, and risperidone. Notably, within three days of the initiated therapy, the patient exhibited significant improvement in terms of reduced agitation, coherent speech, and the development of self-reflection regarding the episode, ultimately leading to his discharge on the seventh day of hospitalization. This case report serves to highlight the complexity of psychiatric presentations associated with underlying endocrine disorders and underscores the importance of interdisciplinary collaboration in achieving optimal patient outcomes.

**Conclusions:**

While mental health factors play a significant role in the development of psychosis, it is essential to recognize that underlying medical conditions may also contribute to its onset or exacerbation.

**Disclosure of Interest:**

None Declared

